# Effect of alkaline fusion on muscovite decomposition and the vanadium release mechanism from vanadium shale

**DOI:** 10.1098/rsos.180700

**Published:** 2018-10-17

**Authors:** Xingwen Jian, Jing Huang, Zhenlei Cai, Yimin Zhang, Tao Liu, Hong Liu

**Affiliations:** 1 School of Resource and Environmental Engineering, Wuhan University of Science and Technology, Wuhan 430081, Hubei Province, People's Republic of China; 2 State Environmental Protection Key Laboratory of Mineral Metallurgical Resources Utilization and Pollution Control, Wuhan University of Science and Technology, Wuhan 430081, Hubei Province, People's Republic of China; 3 Hubei Provincial Engineering Technology Research Center of High Efficient Cleaning Utilization for Shale Vanadium Resource, Wuhan University of Science and Technology, Wuhan 430081, Hubei Province, People's Republic of China; 4 School of Resource and Environmental Engineering, Hubei Collaborative Innovation Center for High Efficient Utilization of Vanadium Resources, Wuhan 430081, Hubei Province, People's Republic of China; 5 School of Resource and Environment Engineering, Wuhan University of Technology, Wuhan 430070, People's Republic of China

**Keywords:** vanadium extraction, vanadium shale, alkaline fusion, muscovite

## Abstract

In order to figure out the decomposition of muscovite and the release mechanism of vanadium from vanadium shale in the alkaline fusion process, the process of vanadium release and roasting kinetics by alkaline fusion was studied. It was found that the addition of sodium hydroxide made the muscovite convert into the sodium silicate and gehlenite. This process promoted the dissolution of silicon and the destruction of muscovite, which could facilitate the release of vanadium. The kinetic analysis indicated that the controlling step of vanadium transformation reaction is changed from chemical reaction control to diffusion control with the increase of roasting time. Compared to the diffusion controlling step, the vanadium related chemical reaction was completed in the first period. The alkaline fusion reaction enhanced the decomposition of muscovite, which could accelerate the release of vanadium and reduce the dependence on high temperature and time in the roasting process. The apparent activation energies of chemical reaction control and diffusion control were 42.24 kJ mol^−1^ and −9.553 kJ mol^−1^, respectively. The kinetic model of vanadium extraction from vanadium shale using alkaline fusion could be finally established.

## 1. Introduction

Vanadium plays significant roles in many fields (such as ferrous and nonferrous alloy production, catalysts and redox flow batteries) because of its good properties [1–3]. More than 87% of the vanadium resources exist as vanadium shale in China [4,5]. Moreover, most of the vanadium in vanadium shale exists as V(III), which substitutes for Al(III) in the dioctahedron of muscovite-group minerals as isomorphism [6,7]. The vanadium in muscovite is always very stable and difficult to extract. In this case, substantial attention has been given the structural breakdown of muscovite in vanadium shale.

Direct acid leaching and roasting-leaching processes are the main technologies for extracting vanadium from vanadium shale and have been widely adopted in recent years. However, there are some problems with the direct acid leaching process [8–10]. First, inorganic acids, especially sulfuric acid, may cause further environmental problems, such as the waste acid solutions generation and toxic fumes. Second, the most important problem is that the sulfuric acid leaching process lacks effectively break the V-bearing lattice of mica minerals, resulting in a lower vanadium leaching efficiency.

Based on the developments of the roasting-leaching technologies, although the sodium roasting-water leaching process was effective for vanadium extraction from vanadium shale, it had significantly adverse effects on the environment because of serious poisonous gas emissions [11,12]. The effect of the calcified roasting-acid leaching process was limited because of the problem associated with high temperature salt roasting technologies [13,14]. Recently, a series of novel composite roasting additive-acid leaching processes have been developed by researchers from our research group [15,16]. However, their industrial application and feasibility are still to be further researched.

As an effective and environmentally friendly technique, the use of alkaline fusion in the mineral and metallurgy fields is increasingly attracting attention. Because of the eco-friendliness, good selectivity and good availability, it has been applied for the recovery of REEs, titanium and silicon from secondary resources [17–19]. Recently, some research has proved that the alkaline fusion of vanadium shale as a pretreatment process makes it possible to increase the vanadium leaching efficiency [20]. However, further investigations on the vanadium extraction mechanisms, based on the intensive systematic discussion of the experimental conditions, are definitely required to explain and promote the application of the alkaline fusion technology.

Therefore, this work is aiming to analyse the decomposition of muscovite and the phase evolution of vanadium in vanadium shale alkaline fusion reaction. The lattice bonding structure and the particle morphology were employed to explain the mechanism of muscovite structure collapse and vanadium release mechanism in vanadium shale by alkaline fusion.

## 2. Experimental set-up

### 2.1. Materials

The raw vanadium shale used in the study was obtained from Tengda Mining Co. Ltd. in Tongshan county, Hubei province, China. The raw vanadium shale was crushed to a grain size of 0–3 mm by a jaw crusher and a double-roll crusher. The crushed ore was subjected to carbon removal pre-treatment by blank roasting in a muffle furnace at 700°C at the rate of 10°C min^−1^ for 60 min and then the ore was ground into powder with the particle size of −0.074 mm by a vibration mill, accounting for 70% of the total. The obtained ore is referred to as a blank roasted sample throughout this study.

The ICP-AES analysis of the raw vanadium shale and blank roasted sample are shown in table 1. It can be seen from the blank roasted sample that the V_2_O_5_ content increased to 0.82% when compared with the raw ore.

**Table 1.** Main chemical composition of raw ore and decarburized sample (wt%).

element	V_2_O_5_	SiO_2_	Al_2_O_3_	CaO	K_2_O	Na_2_O	TFe	C	S
raw vanadium shale	0.70	59.60	8.40	5.01	2.24	0.45	4.13	11.98	3.38
blank roasted sample	0.82	64.80	11.83	5.34	2.68	0.67	4.27	1.28	0.80

### 2.2. Experimental procedures and analytical methods

Here, 30 g of blank roasted sample was mixed with NaOH, according to the 0 to 1 : 1.2 g g^−1^ mass ratio. When the crucible was placed in a muffle furnace, the alkaline fusion was started at different roasting temperatures from 300 to 700°C and different roasting times from 15 to 90 min. At the end of the roasting process, the roasted product was transferred to a leaching pod. The leaching process was carried out at a leaching temperature of 60°C, leaching time of 1 h and a liquid–solid ratio of 4.0 ml g^−1^. After the leaching solution was filtrated, the vanadium leaching efficiency was calculated to evaluate the consequence of the alkaline fusion technology. The vanadium leaching efficiency was calculated as follows:

2.1 $$\eta =\frac{V\beta }{m\alpha }\times 100\text{\%},$$

where *η* is the leaching efficiency of vanadium (wt%), *V* is the volume of the leaching solution (ml), *β* is the content of vanadium in the leaching solution (g ml^−1^), *m* is the mass of the blank roasted sample of vanadium shale (*g*) and *α* is the content of vanadium in the blank roasted sample of vanadium shale (wt%).

The analytical methods were as follows:

1. Chemical compositions were determined by an inductively coupled plasma-atomic emission spectroscope (ICP-AES, Optima-4300DV, PerkinElmer, Boston, MA, USA).
2. Phase compositions were identified by X-ray diffraction (XRD, D/MAX 2500PC, Rigaku, Tokyo, Japan) using Cu K*α* radiation.
3. The bonding structures of muscovite in the alkaline fusion samples were studied by a Fourier infrared (FTIR) spectrometer (Nexus, Thermo Nicolet, USA).
4. Microscopic observation and elemental analysis were conducted with a scanning electron microscope (SEM, JSM-IT300, JEOL, Tokyo, Japan) equipped with an energy dispersive spectrometer (EDS, X-Act, Bruker, Oxford, London, Britain).

## 3. Results and discussion

### 3.1. Alkaline fusion experiments

#### 3.1.1. Effect of mass ratio of NaOH to blank roasted sample on vanadium leaching efficiency

**Figure 1.**
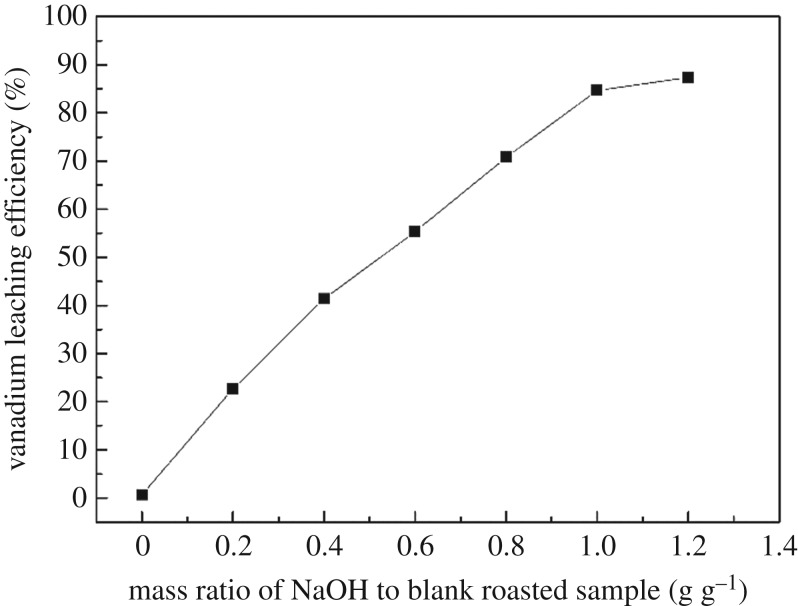
Effect of mass ratio of NaOH to blank roasted sample on vanadium leaching efficiency.

It can be seen from figure 1 that the mass ratio of NaOH to blank roasted sample significantly influenced the vanadium leaching efficiency. When the mass ratio of NaOH to blank roasted sample varied from 0 to 1 : 1 g g^−1^, the vanadium leaching efficiency was 0.688% and 84.63%, respectively. The vanadium leaching efficiency observed to be nearly constant as the mass ratio of NaOH to blank roasted sample exceeded 1 : 1 g g^−1^. Therefore, the mass ratio of NaOH to blank roasted sample at 1 : 1 g g^−1^ was selected as the optimal condition.

#### 3.1.2. Effect of roasting temperature and roasting time on vanadium leaching efficiency

**Figure 2.**
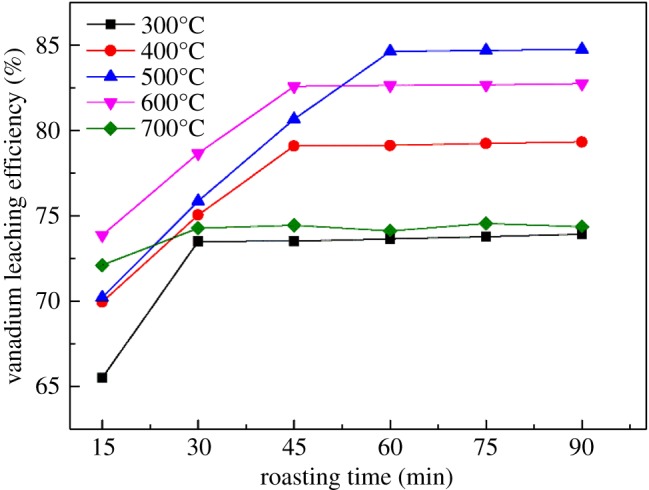
Effect of roasting temperature and roasting time on vanadium leaching efficiency.

It can be seen from figure 2 that the vanadium leaching efficiency increased markedly from 71.75% to 84.63% as the roasting temperature increased from 300 to 500°C for 60 min. However, when the roasting temperature exceeded 500°C, the vanadium leaching efficiency decreased because of the sintering of the materials. Some of the minerals in the vanadium shale reacted to generate glass-like materials and a complex silicate with a low melting point. These reactants wrapped the particles and impeded the transport of vanadium into the leaching solution [20,21]. Hence, the optimum roasting temperature should be 500°C, and the optimum roasting time should be 60 min.

According to the alkaline fusion experiments, the vanadium leaching efficiency observed to be nearly constant of 84.63% as the mass ratio of NaOH to blank roasted sample exceeded 1 : 1 g g^−1^, thus indicating that the roasting process had already reached the optimum influence on vanadium leaching efficiency. Therefore, the phase transformation for gehlenite during the water leaching process has an important impact on the vanadium leaching efficiency. The colloid, which was produced by gehlenite during the water leaching process, covered the surface of the muscovite and thus decreased the leaching efficiency of V [20].

### 3.2. Mechanism analysis of vanadium shale during the alkaline fusion

#### 3.2.1. Chemical phase transformation analysis

**Figure 3.**
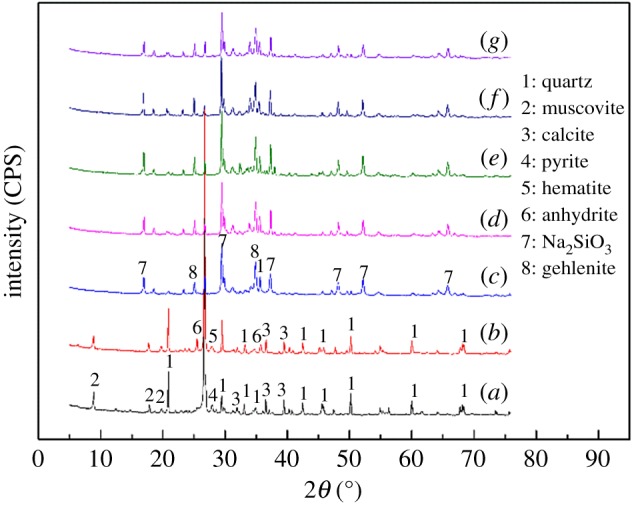
XRD patterns of alkaline fusion samples at different roasting temperature, the blank roasted sample and the raw ore. (*a*) Raw ore; (*b*) blank roasted sample; (*c*) roasting at 300°C; (*d*) roasting at 400°C; (*e*) roasting at 500°C; (*f*) roasting at 600°C; (*g*) roasting for at 700°C.

This analysis is carried out in order to obtain a primary acquaintance of chemical phases transformations during the alkaline fusion process. The XRD patterns of the vanadium shale alkaline fusion products at different roasting temperature, the blank roasted sample and the raw ore are shown in figure 3. It can be seen that the main mineral phases of the raw ore are quartz, muscovite, pyrite and calcite. The results of blank roasted sample when compared with those of the raw ore showed that the phase of pyrite was converted into haematite by roasting in an oxidizing atmosphere, producing SO_2_. Meanwhile, partial calcite was converted into the anhydrite with O_2_ and SO_2_. Moreover, a significant disappearance of muscovite peaks intensity from blank roasted sample to alkaline fusion products was clearly observed, thus indicating that the muscovite structures were thoroughly disintegrated when sodium hydroxide was introduced into the roasting process. It was worth noting that there were two new chemical phases of sodium silicate and gehlenite observed in the XRD patterns of the alkaline fusion samples, indicating that the appearance of new chemical phases was due to the chemical reaction between muscovite and molten sodium hydroxide. In this case, the muscovite structures were disintegrated and vanadium was liberated from the crystal. A small but obvious enhancement of the gehlenite phase could also be observed from 300 to 500°C; it became weaker as the temperature increased, but could also be observed through the whole alkaline fusion reaction, thus indicating that with the further increase of temperature, the reaction of alkaline fusion might have changed and thus prevented the effective recovery of vanadium from the muscovite. The XRD analysis was consistent with the results of the roasting experiments.

#### 3.2.2. Thermodynamic analysis

**Figure 4.**
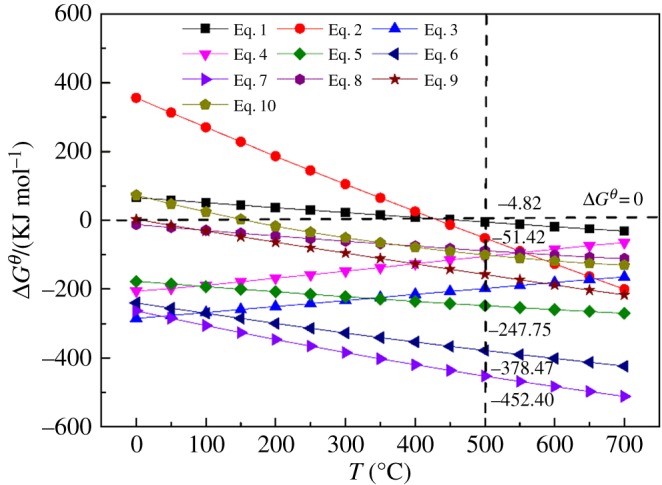
Standard Gibbs free energy changes for reactions.

The thermodynamic analysis was theoretically researched to analyse the chemical reaction of the alkaline fusion, especially the feasibility of muscovite decomposition. When the vanadium shale was roasted without sodium hydroxide, the roasting process could be summarized as equation (2.1), and as shown in table 2 the muscovite disintegrated and released KAlSi_3_O_8_ and Al_2_O_3_. Analyses of the vanadium shale alkaline fusion products suggested that the alkaline fusion process with sodium hydroxide can be described as equation (3.1): sodium hydroxide reacted with muscovite and then generated Na_2_SiO_3_ and gehlenite. V(III) and V(IV) were easily oxidized into the V(V) by O_2_ from the air, represented by equations (3.2) and (3.3). In the alkaline fusion environment, vanadium ions united with sodium ions and formed sodium vanadate, rapidly. Herein, equations (3.4)–(3.6) are the three ultimate reactions generating sodium vanadate, so are equations (3.7)–(3.9) for calcium vanadate. In this work, the standard Gibbs free energy changes Δ*G^θ^* for these reactions at different temperatures could be obtained by the ‘Reaction’ module of FactSage v.7.1 software [21] as shown in figure 4.

**Table 2.** The possible chemical reactions during roasting process.

no.	chemical reactions
1	KAl_2_(AlSi_3_O_10_)(OH)_2_ = KAlSi_3_O_8_+Al_2_O_3_ + H_2_O(g)
2	2KAl_2_(AlSi_3_O_10_)(OH)_2_ + 6NaOH + 6CaCO_3_ = 3Ca_2_Al_2_SiO_7_ + 3Na_2_SiO_3_ + K_2_CO_3_ + 5CO_2_ + 5H_2_O(g)
3	V_2_O_3_ + O_2_ = V_2_O_5_
4	2 V_2_O_4_ + O_2_ = 2 V_2_O_5_
5	2NaOH + V_2_O_5_ = 2NaVO_3_ + H_2_O(g)
6	4NaOH + V_2_O_5_ = Na_4_V_2_O_7_ + 2H_2_O(g)
7	6NaOH + V_2_O_5_ = 2Na_3_VO_4_ + 3H_2_O(g)
8	CaCO_3_ + V_2_O_5_ = CaV_2_O_6_ + CO_2_(g)
9	2CaCO_3_ + V_2_O_5_ = Ca_2_V_2_O_7_ + 2CO_2_(g)
10	3CaCO_3_ + V_2_O_5_ = Ca_3_V_2_O_8_ + 3CO_2_(g)

**Figure 5.**
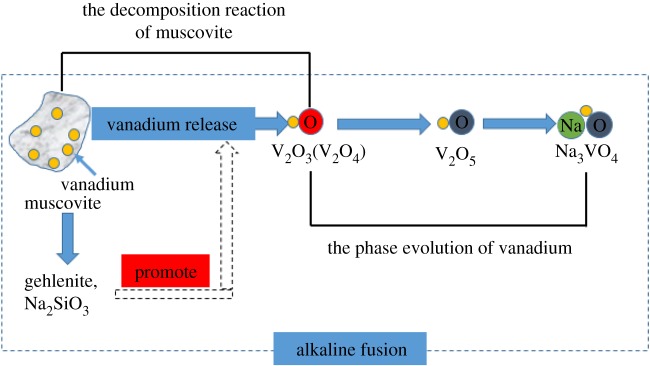
The phase evolution of muscovite to sodium vanadate during alkaline fusion.

It can be seen in figure 4 that the Δ*G^θ^* (500°C) values were negative for all reaction equations, indicating that equations (2.1), (3.1)–(3.9) were spontaneous at 500°C during alkaline fusion. V(III) and V(IV) could be oxidized to V(V) during the roasting process, and V_2_O_5_ would react with other oxides to form vanadate. The Δ*G^θ^* (500°C) value for equation (3.1) was more negative than equation (2.1), indicating that the decomposition reaction of muscovite during alkaline fusion was easier than the blank roasting process. The Δ*G^θ^* (500°C) values for equations (3.4)–(3.6) were more negative than equations (3.7)–(3.9), indicating that the formation of sodium vanadate was easier than calcium vanadate during the alkaline fusion process. Thus, the Gibbs free energy of muscovite decomposition reaction was reduced by alkaline fusion. So, the decomposition reaction of muscovite can be carried out at a lower temperature, the extent of destruction in muscovite structure increases and more V(III) was liberated from the crystal lattice and oxidized into V(IV) or V(V). Meanwhile, the formation of sodium vanadate was promoted with the generation of calcium vanadate inhibited during alkaline fusion. As a result, the vanadium leaching efficiency was enhanced by alkaline fusion. Therefore, the phase evolution of muscovite to sodium vanadate took place in the following sequential order (figure 5).

#### 3.2.3. Bonding structure of muscovite in alkaline fusion samples

**Figure 6.**
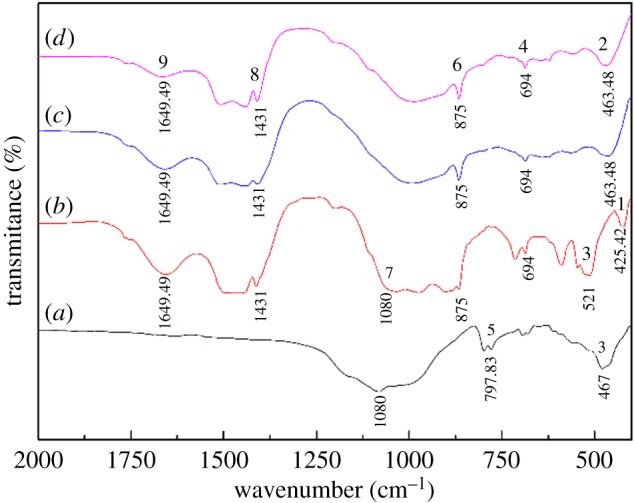
FTIR spectra of alkaline fusion samples at different roasting temperature and blank roasted sample. (*a*) Blank roasted sample; (*b*) roasting at 300°C; (*c*) roasting at 400°C; (*d*) roasting at 500°C.

Figure 6 shows the FTIR analysis of the vanadium shale alkaline fusion samples at 300 to 500°C and the blank roasted sample. Band 1(close to 425.42 cm^−1^) and Band 2(close to 436.48 cm^−1^) is attributed to O–Si(Al)–O bending vibrations of feldspar minerals. Band 3 (close to 467 and 521 cm^−1^) is attributed to Si–O–Al bending vibrations of muscovite overlapped with Si–O bending vibrations of quartz [22]. Band 4 (close to 694 cm^−1^) and Band 5 (close to 797.83 cm^−1^) are attributed to Si–O stretching vibrations of sodium silicate and quartz. Band 6 (close to 875 cm^−1^) is attributed to C–O stretching vibrations of carbonate. Band 7 (close to 1029 cm^−1^) is attributed to Si(Al^IV^)–O stretching vibrations of the tetrahedral layer of muscovite [23]. Band 8 (close to 1431 cm^−1^) and Band 9 (close to 1649.49 cm^−1^) are attributed to O–H stretching vibrations and flexural vibrations of the sodium hydroxide. It can be seen that band 3 divided rapidly to form bands 1 and 2, and band 7 gradually decreases in intensity and then disappears as the temperature increases, indicating that the molten sodium hydroxide assisted the breakdown of muscovite structure, which gives rise to a further increase in the release of vanadium. Meanwhile, the change between bands 5 and 6 implies the conversion of quartz to sodium silicate. The appearance of bands 8, 9 and band 6 indicate that there may have an excess of sodium hydroxide in the alkaline fusion products. In general, the main changes in the spectra of the alkaline fusion samples occur at bands 3 and 7 and reflect subtle changes in the muscovite structure during the roasting processes.

#### 3.2.4. Surface morphology analysis

**Figure 7.**
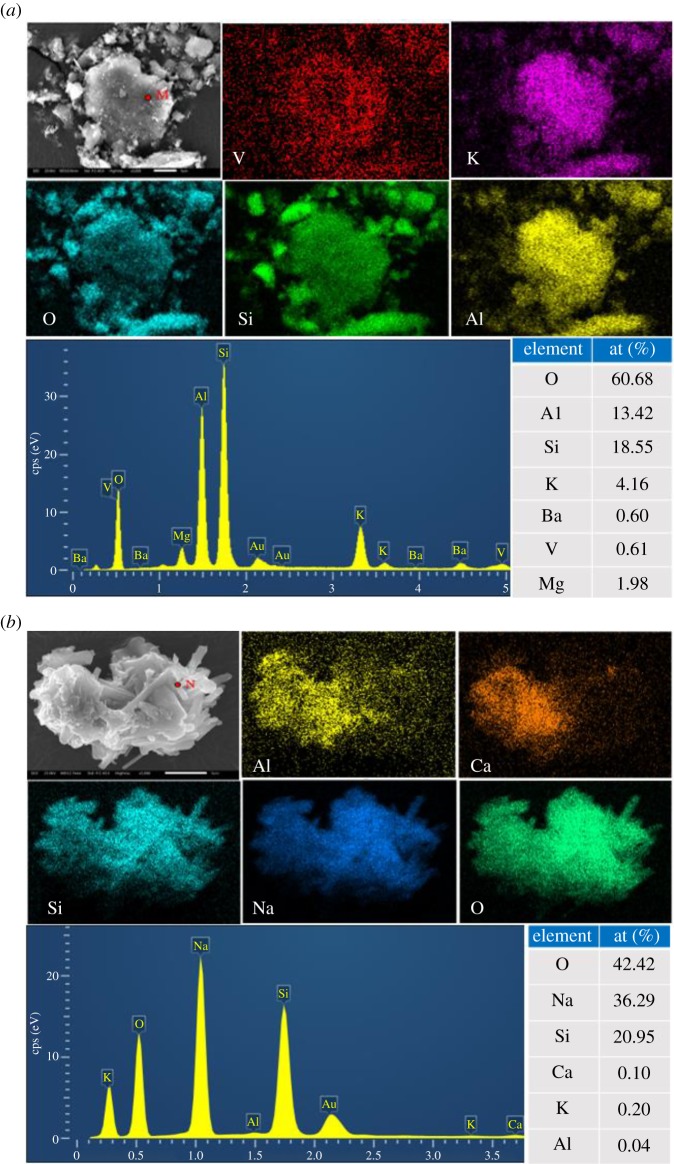
SEM micrographs with EDS element mapping of alkaline fusion sample at 500°C and blank roasted sample. (*a*) SEM micrograph with EDS element mapping of blank roasted sample. (*b*) SEM micrograph with EDS element mapping of alkaline fusion sample at 500°C.

In order to obtain a visualized insight of the behaviours of the vanadium occurrence state during alkaline fusion processes, SEM-EDS analysis was conducted and the result is presented in figure 7. The SEM micrograph with EDS element mapping of blank roasted sample is shown in figure 7*a*, the clear profile and denseness implying that the relevance of V, K, O, Si and Al was good, and the K, O, Si and Al atom percentages at point ‘M’ were close to those of muscovite, proving that V still existed in the muscovite. As for the SEM micrograph with EDS element mapping of alkaline fusion sample at 500°C shown in figure 7*b*, it is clearly seen that not only the Na, Si and O, but also the Ca, Al, Si and O had obvious relevance. Combined with the XRD and thermodynamic analyses, gehlenite and sodium silicate indeed existed after the alkaline fusion process, indicating that the muscovite may produce new substances during alkaline fusion. In summary, the differences in the surface morphology and the element relevance between blank roasted sample and the alkaline fusion products indicate that the structures of muscovite have encountered devastating destruction with the formation of new substances. The EDS spectra analysis of the ‘N’ point in figure 7*b* showed that the sodium content was 36.29%, which was close to the theoretical sodium content 37.70% of the sodium silicate (Na_2_SiO_3_). Based on the specific phase transformation, conceivable chemical reaction, clear change of lattice bonding structure and visualized insight of the behaviours of the muscovite during alkaline fusion processes, it can be concluded that the transformation of muscovite converts into the sodium silicate and gehlenite, furthering dissolution of silicon, generating expansion and crack to muscovite, which facilitates the release of vanadium.

### 3.3. Kinetic analysis of vanadium shale during the alkaline fusion reaction

#### 3.3.1. Calculation of reaction orders

At present, the models used in the research of the kinetics of the roasting process are the nucleation reaction model and the regional reaction model [24–26], etc. These models are derived from the assumption that the roasted material is formed of spherical particles, but the vanadium-containing carrier in the vanadium shale alkaline fusion system is the flaky mica minerals. These mineral particles exist as chemical heterosexual during the roasting process. Therefore, the traditional shrinking reaction model and the regional reaction model cannot exactly describe the roasting process of flake structured minerals. So, the experimental data could be analysed using the dynamic model of the flaky structure mineral roasting process [27,28].

**Figure 8.**
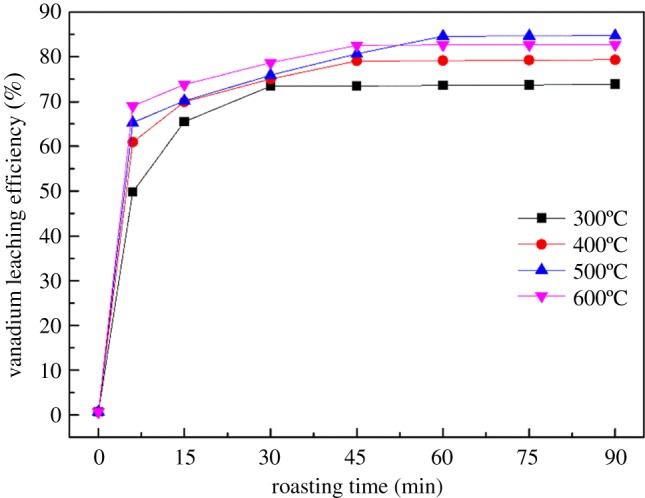
Relationships between vanadium transforming efficiency and roasting time with different roasting temperature.

To analyse the reaction rate during alkaline fusion, the roasting kinetic analysis was theoretically investigated. For the roasting-water leaching technology, a series of chemical reactions of vanadium have been examined to occur in the roasting process, and the dissolution of vanadate was the main action in the process of water leaching [29,30]. Therefore, the transformation efficiency of vanadium in the roasting process can be used to evaluate the roasting effect. In order to draw the kinetic curve of alkaline fusion reaction, figure 8 presents the effects of different roasting temperatures (from 300 to 600°C) and different roasting times (from 6 to 90 min) on vanadium transforming efficiency. The most appropriate mass ratio of NaOH to blank roasted sample was 1 : 1 g g^−1^, which was based on the previous alkaline fusion experiments. It can be seen that the vanadium transforming efficiency was increased with the increase of the roasting temperature during the overall alkaline fusion experiments. The vanadium transforming efficiency was observed to be decreasing as the roasting temperature exceeded 500°C, which was the result of a sintering phenomenon.

As for the vanadium shale alkaline fusion reaction, if the process is controlled by the chemical reaction, the following expression of the dynamic model of flaky structure mineral could be used to describe the kinetics of the process:

3.1 n=1F(G)=−ln⁡(1−G+g)=kt

and

3.2 $$n\neq 1\;F(G)=\frac{1}{1-n}-{\frac{\left(1-G+g\right)}{1-n}}^{1-n}=kt.$$

Similarly, when the diffusion of the reactants or products through a product layer is the controlling step, the following expression of the dynamic model of flaky structure mineral could be used:

3.3 $$F(G)={\left(G-g\right)}^{2}=kt,$$

where *G* is the vanadium transforming efficiency; *t* is the reaction time; *n* is the reaction order; *k* is the velocity constant; *g* is the conversion rate of vanadium in blank roasted process, which in this study has been proved to be 0.688% by the blank roasted direct water leaching experiment, in which the blank roasted sample had a leaching temperature of 60°C, a leaching time of 1 h and a liquid–solid ratio of 4.0 ml g^−1^.

It can be seen from figure 8 that the alkaline fusion process was obviously divided into two stages. In the first period of the roasting time, the vanadium transforming efficiency increased rapidly; however, further extension of the roasting time had not improved the vanadium transforming efficiency. Therefore, it can be concluded that the rate-controlling steps of the alkaline fusion process were the diffusion through and the surface chemical reaction and the solid product layer, respectively, during the alkaline fusion process.

**Figure 9.**
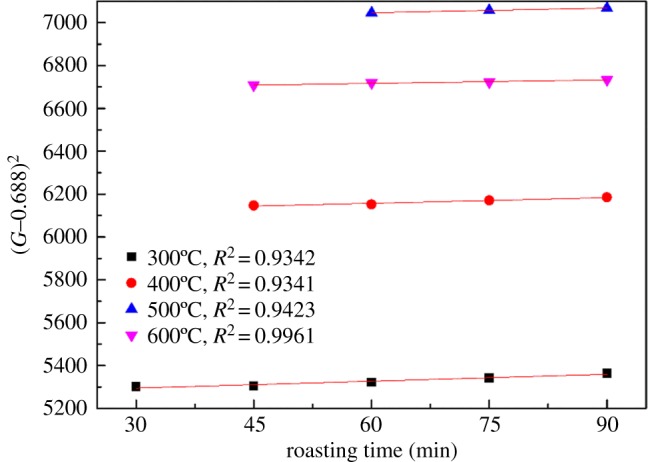
Fitting plot of (*G*−0.688)^2^ versus time at different reaction temperatures.

Based on the experimental results provided in figure 8, the results of the linear fitting data on alkaline fusion reaction controlled by diffusion is shown in table 3. The kinetics equation of diffusion control is relatively simple. Therefore, the matching analysis of the diffusion control kinetic equation is carried out. It is known that the square of the transforming rate (*G*−0.688)^2^ is linearly related to the reaction time *t* if the roasting process is controlled by diffusion. Figure 9 shows the relationship between (*G*−0.688)^2^ and *t* during alkaline fusion. During the reaction time shown in figure 9, the data are linear, which indicates that the rate of reaction is controlled by diffusion through the product layer during that part of the roasting time.

**Table 3.** Linear fitting data on alkaline fusion controlled by diffusion.

	controlled by diffusion		
temperature (°C)	time (min)	*k* (min^−1^)	*R*^2^
300	30–90	1.090	0.9342
400	45–90	0.882	0.9341
500	60–90	0.714	0.9423
600	45–90	0.533	0.9961

The kinetics equation of flake structure minerals controlled by a chemical reaction (equations (3.1) and (3.2)) show that the reaction order *n* should be determined in order to investigate whether the chemical reaction is controlled in the early stage of roasting. The differential method is used to determine the reaction order, for which the following expression could be used:

3.4 $$\ln\frac{dG}{\mathrm{dt}}=\ln(K_{n}\cdot 2ab)+n\ln(1-G),$$

where *G* is the vanadium transforming efficiency; *t* is the reaction time; *n* is the reaction order; *K_n_* is the velocity constant; *a* and *b* are the length and width of reactant, respectively.

Based on the experimental results provided in figure 8, the results of the linear fitting data on the alkaline fusion reaction controlled by chemical reaction is shown in table 4.

**Table 4.** Linear fitting data on alkaline fusion controlled by chemical reaction.

	controlled by chemical reaction				
temperature (°C)	time (min)	*n*	*R*^2^	*k* (min^−1^)	*R*^2^
300	0–30	4.2889	0.9145	1.15×10^−4^	0.9681
400	0–45	3.5755	0.9348	7.40×10^−4^	0.9950
500	0–60	3.1565	0.9101	8.38×10^−4^	0.9931
600	0–45	3.0926	0.9644	3.19×10^−3^	0.9826

As shown in table 4, the reaction order at 300°C is 4.2889, the equation can be expressed as follows:

3.5 $$F(G)=-0.304+0.304{\left(100.688-G\right)}^{-3.2889}=kt.$$

The reaction order at 400°C is 3.5807, the equation can be expressed as follows:

3.6 $$F(G)=-0.387+0.387{\left(100.688-G\right)}^{-2.5807}=kt.$$

The reaction order at 500°C is 3.1565, the equation can be expressed as follows:

3.7 $$F(G)=-0.464+0.464{\left(100.688-G\right)}^{-2.1565}=kt.$$

The reaction order at 600°C is 3.0926, the equation can be expressed as follows:

3.8 $$F(G)=-0.478+0.478{\left(100.688-G\right)}^{-2.3352}=kt.$$

**Figure 10.**
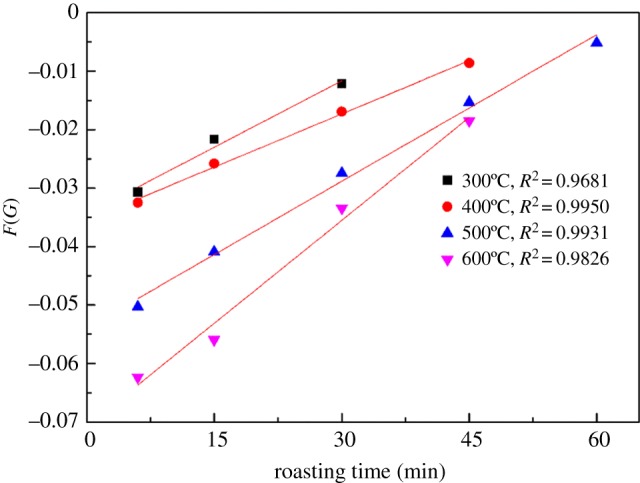
Fitting plot of *F*(*G*) versus time at different reaction temperatures.

According to these results provided in equations (3.5)–(3.8), figure 10 gives a fitting plot of *F*(*G*) versus *t*. From the slopes of the fitting lines in figure 10, the apparent rate constant (*k*) values were determined. It can be observed that the linear correlation was great during these alkaline fusion reaction stages, indicating that the alkaline fusion reaction of these roasting stages was controlled by the chemical reaction.

#### 3.3.2. Calculation of apparent activation energy

**Figure 11.**
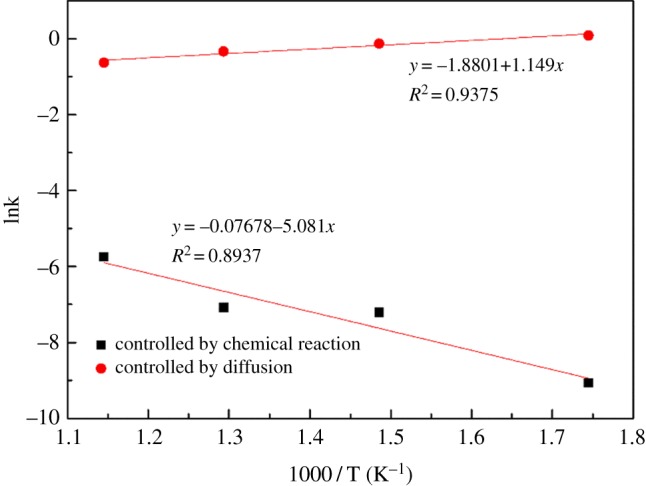
Arrhenius fitting plot of ln *k* as a function of 1/(1000 T) during alkaline fusion.

The effect of roasting temperature on vanadium transforming efficiency results was analysed by using the dynamic model of flaky structure mineral to calculate the kinetic parameters. Figure 11 shows the detailed results of the activation energy, *E*_a_, and the coefficients of determination (*R*^2^).

According to the slopes of the fitting lines in tables 3 and 4, the apparent rate constant (*k*) values were obtained and a fitting plot of ln *k* versus 1/(1000 T) is shown in figure 11. With the slope of the fitting line above, the apparent activation energy (*E*_a_) for chemical reaction control and diffusion control stage could be calculated as 42.24 and −9.553 kJ mol^−1^ by the Arrhenius equation as follows:

3.9 $$\ln k=-\frac{Ea}{R}\times \frac{1}{T}+\ln A,$$

where *k* is the reaction rate constant, *A* is the pre-exponential factor, *E*_a_ is the apparent activation energy (kJ mol^−1^), *R* is the gas constant (J mol^−1^· K), *T* is the roasting temperature (K).

In summary, it can be concluded from the kinetics analysis that the alkaline fusion process was controlled by chemical reaction in the first period, vanadium was oxidized and transformed in this process. It can be concluded that alkaline fusion can accelerate the release of vanadium and reduce the dependence on high temperature and time in the roasting process. The apparent activation energy of the diffusion control stage during the roasting process is negative, indicating that as the temperature increases, the product's diffusion rate decreases, which may be due to the addition of sodium hydroxide, and sintering occurs at high temperatures which hinders the diffusion of the product. The variation tendency of the calculated activation energy clearly shows that the reaction of the vanadium shale alkaline fusion is a multi-step process. This result agrees with the reaction mechanism mentioned above.

## 4. Conclusion

The alkaline fusion process had an efficient extraction of vanadium from the vanadium shale though drawing molten alkali to produce local deformation of muscovite particles for a complete decomposition of the muscovite. The transformation of muscovite to the Na_2_SiO_3_ and gehlenite enhanced the defects of muscovite particles, boosting dissolution of silicon, generating expansion and cracking to muscovite particles. Thus, the alkaline fusion samples exhibited more cracks and their particles were more porous, which could promote the liberation of vanadium.

The vanadium shale alkaline fusion kinetic analysis showed that the early stage of the roasting process is controlled by chemical reaction, but the reaction process transformed into diffusion control quickly. The alkaline fusion method could significantly enhance the decomposition reaction of muscovite, which can accelerate the release of vanadium and reduce the dependence on high temperature and time in the roasting process. The apparent activation energy could be achieved as 42.24 kJ mol^−1^ and −9.553 kJ mol^−1^, respectively. The variation tendency of the calculated activation energy was consistent with the proposed reaction mechanism.

## Supplementary Material

The vanadium leaching efficiency

## Data accessibility

A detailed experimental section can be found in the electronic supplementary material.
