# Supplementary material for: Effect of alkaline fusion on muscovite decomposition and the vanadium release mechanism from vanadium shale
Source: R Soc Open Sci. 2018 Oct 17;5(10):180700. doi: 10.1098/rsos.180700 (PMC6227943; doi:10.1098/rsos.180700)
Supplement: The vanadium leaching efficiency [file rsos180700supp1.docx]

The detailed experimental data of **Fig. 1**

| mass ratio of NaOH to blank roasted sample | vanadium leaching efficiency |
| --- | --- |
| 0 | 0.688% |
| 0.2 | 22.62% |
| 0.4 | 41.41% |
| 0.6 | 55.33% |
| 0.8 | 70.77% |
| 1.0 | 84.63% |
| 1.2 | 87.38% |

The detailed experimental data of **Fig. 2**

| roasting temperature  roasting time | 300°C | 400°C | 500°C | 600°C |
| --- | --- | --- | --- | --- |
| 15 min | 65.51% | 69.95% | 70.21% | 73.86% |
| 30 min | 73.50% | 75.05% | 75.87% | 78.66% |
| 45 min | 73.52% | 79.09% | 80.66% | 82.59% |
| 60 min | 73.64% | 79.12% | 84.63% | 82.66% |
| 75 min | 73.78% | 79.24% | 84.70% | 82.68% |
| 90 min | 73.93% | 79.33% | 84.76% | 82.75% |

vanadium leaching efficiency

The detailed experimental data of **Fig. 8**

| roasting temperature  roasting time | 300°C | 400°C | 500°C | 600°C |
| --- | --- | --- | --- | --- |
| 0 min | 0.688% | 0.688% | 0.688% | 0.688% |
| 6 min | 49.81% | 60.92% | 65.30% | 69.02% |
| 15 min | 65.51% | 69.95% | 70.21% | 73.86% |
| 30 min | 73.50% | 75.05% | 75.87% | 78.66% |
| 45 min | 73.52% | 79.09% | 80.66% | 82.59% |
| 60 min | 73.64% | 79.12% | 84.63% | 82.66% |
| 75 min | 73.78% | 79.24% | 84.70% | 82.68% |
| 90 min | 73.93% | 79.33% | 84.76% | 82.75% |

vanadium transforming efficiency
